# Jagged-1+ skin Tregs modulate cutaneous wound healing

**DOI:** 10.1038/s41598-024-71512-1

**Published:** 2024-09-09

**Authors:** Prudence PokWai Lui, Jessie Z. Xu, Hafsah Aziz, Monica Sen, Niwa Ali

**Affiliations:** 1 Peter Gorer Department of Immunobiology, King’s College London, London, SE1 9RT UK; 2 Centre for Gene Therapy and Regenerative Medicine, King’s College London, London, SE1 9RT UK

**Keywords:** Regulatory T cells, Inflammation

## Abstract

Skin-resident regulatory T cells (Tregs) play an irreplaceable role in orchestrating cutaneous immune homeostasis and repair, including the promotion of hair regeneration via the Notch signaling ligand Jagged-1 (Jag1). While skin Tregs are indispensable for facilitating tissue repair post-wounding, it remains unknown if Jag1-expressing skin Tregs impact wound healing. Using a tamoxifen inducible Foxp3^creERT2^Jag1^fl/fl^ model, we show that loss of functional Jag1 in Tregs significantly delays the rate of full-thickness wound closure. Unlike in hair regeneration, skin Tregs do not utilize Jag1 to impact epithelial stem cells during wound healing. Instead, mice with Treg-specific Jag1 ablation exhibit a significant reduction in Ly6G + neutrophil accumulation at the wound site. However, during both homeostasis and wound healing, the loss of Jag1 in Tregs does not impact the overall abundance or activation profile of immune cell targets in the skin, such as CD4+ and CD8+ T cells, or pro-inflammatory macrophages. This collectively suggests that skin Tregs may utilize Jag1-Notch signalling to co-ordinate innate cell recruitment under conditions of injury but not homeostasis. Overall, our study demonstrates the importance of Jag1 expression in Tregs to facilitate adequate wound repair in the skin.

## Introduction

The skin, the largest mammalian organ, acts as a critical physical barrier from constant environmental traumas. A diverse array of immune and non-immune cell types reside in or migrate to the skin during pre- and post-natal development, influenced by the local inflammatory microenvironment. Over the past decade, skin resident regulatory T cells (Tregs), a major T cell population, have been implicated in various processes, including hair regeneration^1,2^, full-thickness wound healing^3^, epidermal barrier repair^4,5^ and fibrosis^6^. These functions are facilitated by the immunosuppressive capacity of skin Tregs, including restriction of pro-inflammatory cytokine production and myeloid cell accumulation^3,4^, as well as their interaction with non-immune tissue cells such as epithelial keratinocytes^5^, fibroblasts^6^ and stem cells^1,4^.

Multiple single cell studies have illustrated tissue Tregs are transcriptionally distinct from those within secondary lymphoid organs^7–10^. The site where Tregs are seeded determines their phenotype and shapes their functions^7,10^. Previous RNA sequencing analysis identified Jagged-1 (Jag1), one of the five Notch signalling ligands, as a key transcript preferentially expressed in skin Tregs compared to skin-draining lymph node (SDLN) Tregs^1^. Interestingly, intraperitoneal injection of IL-2/anti-IL2 antibody complexes can selectively expand Jag1 positive (Jag1^Pos^)Tregs in murine skin but not in SDLNs^11^. In silico data indicate that skin Tregs are also phenotypically distinct from other tissue-resident Tregs^6,9,10^*.* Ligand-receptor prediction analyses from scRNA-seq datasets further suggest that skin Tregs likely interact with epithelial cells and hair follicle stem cells via Jag1-induced Notch signalling^2,5^, underscoring the potential importance of Jag1 in skin Tregs. Yet, the question of whether Jag1 expression is indeed unique to skin Tregs remains unanswered.

Notch signalling is crucial for hair follicle differentiation and postnatal maintenance homeostatically^12^, as well as in regulating wound healing^13^. Conditional deletion of Jag1 in epidermal stem cells (K15^cre^Jag1^fl/fl^) delays wound closure, whereas the addition of Jag1 peptide accelerates wound healing^13^, demonstrating the significance of Jag1 in the wound repair process. Although it has been established that Jag1 expressed on non-lymphoid cells can drive T cell fate decisions^14,15^, promote Treg expansion^16–18^*,* and that Notch signalling regulates Treg immunosuppressive capacity^19^, the function(s) of Treg-derived Jag1 remains largely unknown. Previously, we have shown the perturbation of Jag1 in skin Tregs hinders hair follicle stem cell (HFSC) proliferation, delays the induction of the hair growth phase, and ultimately impedes hair regeneration^1^. Whether the relationship between Jag1^Pos^ Tregs and hair regeneration translates to other skin Treg-mediated mechanisms, such as wound healing, remains unexplored.

Here, we report that Jag1 is preferentially expressed in skin Tregs compared to Tregs residing in other tissues. Despite Jag1^Pos^ Tregs displaying higher CTLA4 and CD25 surface expression, the absence of Jag1 in Tregs does not alter skin integrity nor overall cutaneous immune dynamics during homeostasis. However, during full thickness wound healing, mice with Jag1 deficiency in Tregs heal significantly slower than controls. Unlike in hair regeneration, skin Tregs in wounded mice do not utilize Jag1 to alter HFSC activation, but rather promote, neutrophil accumulation at the wound site. This study sheds light on an alternative function of Jag1 in skin Tregs in facilitating adequate cutaneous wound repair.

## Results

### Jagged1 is a skin Treg preferential marker

**Fig. 1:**
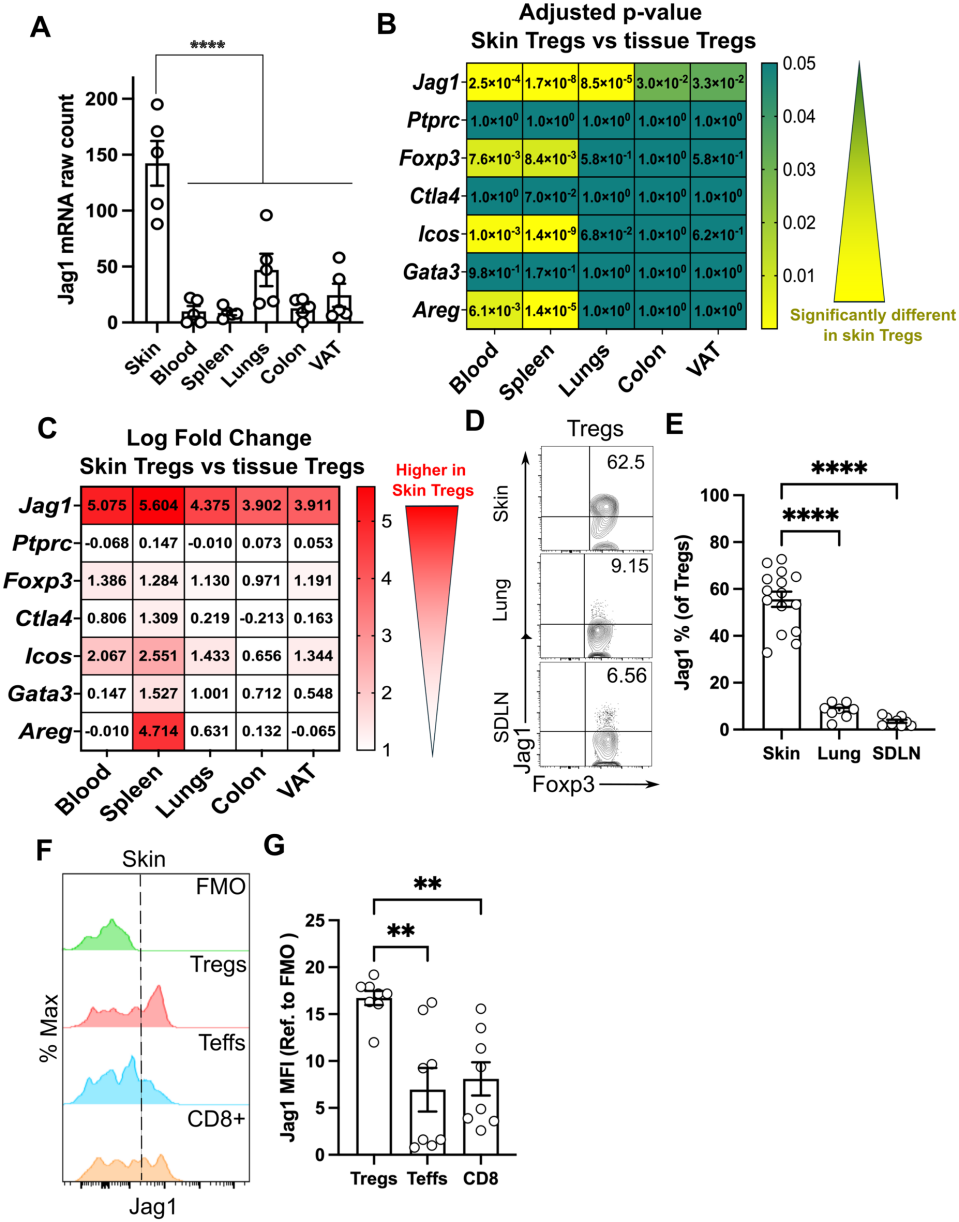
Jagged1 is preferentially expressed in skin Tregs. (**A**) Jag1 mRNA counts from bulk RNAseq of Tregs from different tissue sites. (n = 5). (**B**,**C**) Heatmap showing the adjusted *p*-value (**B**) and log fold change (**C**) of genes expressed in Tregs from skin compared against those from other tissues. (**D**,**E**) Representative flow cytometry plot (**D**) and quantification (**E**) of Jag1^Pos^ Tregs in skin, lung and skin-draining lymph nodes (SDLNs) (n = 8–15). (**F**,**G**) Representative histogram (**F**) and quantification (**G**) of Jag1 expression in different skin T-cell populations (n = 8). Data in (**D**,**E**) or (**F**,**G**) were pooled from 3 independent experiments. Individual data points are shown and presented as mean ± SEM. Statistics in (**A**), (**E**) and (**G**) were calculated by one-way ANOVA, **p < 0.01, ****p < 0.0001.

To determine whether Jag1 is uniquely expressed in skin Tregs, we began by re-analysing a bulkRNAseq dataset focusing on Tregs from various tissues^10^. Our analysis confirmed that skin Tregs exhibited the highest levels of *Jag1* compared to Tregs from blood, spleen, and other organ tissues (visceral adipose tissue (VAT), lung and colon) (Fig. 1A). Differential expression analysis using edgeR and limma packages normalized counts and compared the adjusted *p*-values (Fig. 1B) and log fold changes (Fig. 1C) of *Jag1* expression in skin Tregs against other tissue Tregs. We included *Cd45* (*Ptprc*) and common Treg markers (*Foxp3*, *Ctla4*, *Cd25*, and *Icos*), along with known tissue Treg-associated transcripts (*Gata3* and *Areg*) as controls.

Notably, *Jag1* expression in skin Tregs was significantly and consistently higher than, not only those in blood or lymphoid organs, but also Tregs residing in lung, colon and VAT (Fig. 1B,C). Skin Tregs expressed an average 4.5-fold higher level of *Jag1* than other surveyed Tregs, clearly distinguishing Jag1 from other Treg-associated genes. As expected, *Cd45 (Ptprc)* showed no significant differences (high adjusted *p*-value and low log fold change) between skin and other tissue Tregs. Skin Tregs expressed similar level of *Foxp3* and common Treg markers, as well as tissue Treg-associated genes, compared to Tregs in the lung, colon, and VAT. While *Ctla4* and *Gata3* expression levels remained similar, *Foxp3* and *Icos* were significantly but mildly upregulated in skin Tregs, when compared to those in blood or spleen. Similar to *Jag1*, *Areg* was highly expressed in skin Tregs compared to splenic Tregs, but not when compared to other tissue Tregs. Collectively, this highlights *Jag1* as a differentially expressed marker in skin Tregs.

To further validate these findings, we performed flow cytometry on Tregs from skin, lung and SDLN. Around 55.59% (± 12.61%) of skin Tregs expressed Jag1, in contrast to only 8.18% (± 3.20%, *p* < 0.0001) in lung Tregs and 3.54% (± 1.90%, *p* < 0.0001) in SDLN Tregs (Fig. 1D,E, gating strategy in Supplementary Fig. 1A). The mean fluorescence intensity of Jag1 was significantly higher in skin Tregs (16.72 ± 2.15), in comparison to other CD4+ Foxp3-T effector (Teffs) (6.95 ± 6.57, *p* = 0.0014) and CD8+ T cells (8.10 ± 5.01, *p* = 0.0041), indicating preferential expression of Jag1 in skin Tregs.

We then examined whether these abundant Jag1^pos^ skin Tregs are phenotypically distinct from Jag1-negative (Jag1^neg^) skin Tregs. In adult mice, Jag1^pos^Tregs showed significantly higher proportions and levels of CTLA4 (Supplementary Fig. 2A–D) and CD25 (Supplementary Fig. 2E–H), but not ICOS (Supplementary Fig. 22I–L). Jag1^pos^ skin Tregs co-expressed CTLA4 and CD25 more frequently than Jag1^neg^ Tregs (Supplementary Fig. 2 M,N, 37 ± 4.64% vs 23.5 ± 7.90%, *p* = 0.0312), suggesting that Jag1^pos^ Tregs may be phenotypically more active.

### Jag1^pos^ Tregs are dispensable during the steady state

**Fig. 2:**
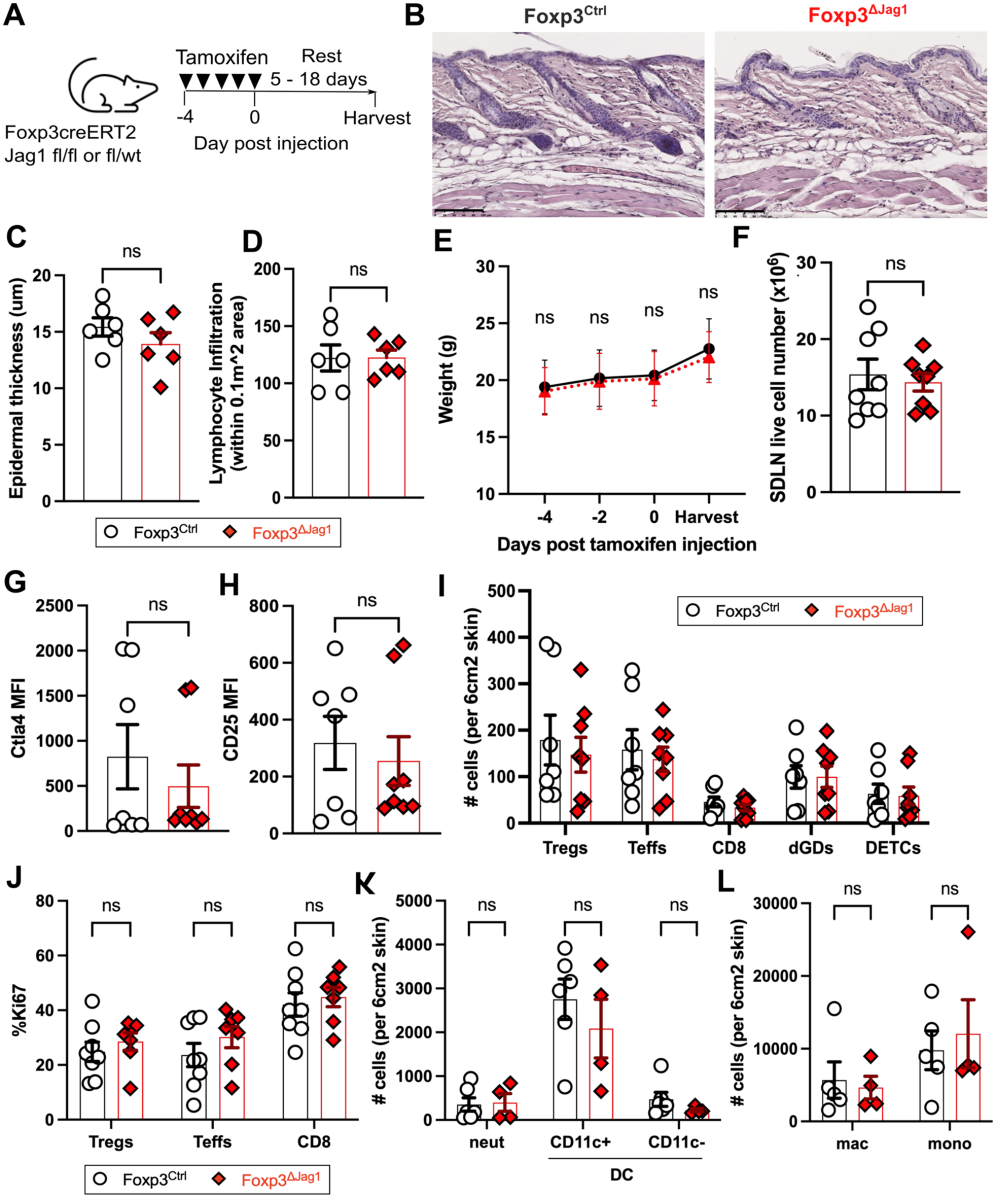
Jag1 in Tregs is dispensable for skin homeostasis. (**A**) Experimental schematic illustrating intraperitoneal injection of tamoxifen daily for 5 days, with 5–18 days rests before harvest for downstream analysis. (**B**) Representative H&E staining of Foxp3^Ctrl^ and Foxp3^ΔJag1^ skin. Scale bars represent 100 μm. (**C**,**D**) Quantification of epidermal thickness (**C**) and lymphocyte infiltration (**D**) from three regions-of-interest in H&E staining of Foxp3^Ctrl^ and Foxp3^ΔJag1^ skin (n = 2 per group)(**E**) Average weight of treated animals traced throughout the course of tamoxifen injection and harvest end point (n = 4 per group). (**F**) Total live cells from Foxp3^Ctrl^ and Foxp3^ΔJag1^ SDLNs (n = 8 per group). (**G**,**H**) Flow cytometric quantification of mean fluorescence intensity (MFI) of CTLA4 (**G**) and CD25 (**H**) in Tregs from Foxp3^Ctrl^ and Foxp3^ΔJag1^ skin (n = 7-8). (**I**,**J**) Quantification of skin T cell abundance (**I**) and proliferation (**J**) in Foxp3^Ctrl^ and Foxp3^ΔJag1^ mice (n = 7-8 per group). (**K**,**L**) Quantification of neutrophils (neut), CD11c + and CD11c-dendritic cells (DCs), macrophages (mac) and monocytes (mono) from wildtype Foxp3^Ctrl^ and Foxp3^ΔJag1^ mice (n = 4–6 per group). Data in (**E**) to (**L**) were harvested from 4 independent experiments collected between 5 and 18 days post last tamoxifen injection. Data in (**B**–**D**) were from 1 and (**K**,**L**) from 2 of these 4 experiments. Each individual data point represent one biological replicate. Results were presented as mean ± SEM. Statistics were calculated by unpaired t-test (**C**,**D**,**F**,**G** and **H**) and two-way ANOVA (**I**–**L**), ns = non-significant.

Under homeostatic conditions, skin Tregs suppress long-term CD8 and Teff cell-driven hair follicle-associated inflammation via CD25^20^. Given that Jag1^pos^ skin Tregs express higher levels of CD25 than Jag1^neg^ skin Tregs, we hypothesised that Jag1^pos^ skin Tregs may be functionally important during the steady state. To test this, and to avoid influencing early skin development, we bred a tamoxifen inducible cell specific model to delete Jag1 in Tregs. This involved crossing mice expressing EGFP-creERT2 gene under the Foxp3 promoter^20–24^ to mice carrying a Jag1 floxed allele with a dysfunctional Delta-Serrate-Lag2 domain of Jag1^25–27^ to generate Foxp3^creERT2^Jag1^fl/fl^ and Foxp3^creERT2^Jag1^fl/wt^, hereafter denoted as “Foxp3^ΔJag1^ “ and “Foxp3^Ctrl^”, respectively. Following intraperitoneal injection of tamoxifen (Fig. 2A), we observed effective downregulation of Jag1 transcript in sorted Tregs from Foxp3^ΛJag1^ compared to Foxp3^Ctrl^ mice (Supplementary Fig. 3A). Expression of *Jag1* in Teffs, CD8+ T cells, and *Foxp3* expression in Tregs (Supplementary Fig. 3B) showed no differences between Foxp3^ΔJag1^ and Foxp3^Ctrl^ animals, indicating Treg-specificity of Jag1 deletion in this model.

Despite systemic loss of Jag1 mRNA in Tregs, the absence of Jag1 did not trigger skin inflammation. H&E staining revealed similar morphology (Fig. 2B), epidermal thickness (Fig. 2C) and lymphocytic infiltration (Fig. 2D) in Foxp3^ΔJag1^ and Foxp3^Ctrl^ animals. Body weight throughout tamoxifen injections (Fig. 2E) and SDLN live cell numbers (Fig. 2F) remained unchanged between animals with or without Jag1 in Tregs during the steady state. Loss of Jag1 in Tregs did not alter overall CD25 or CTLA4 expression in skin Tregs (Fig. 2G,H), nor the abundance and proliferation of skin-resident Tregs, Teff, CD8, gamma-delta T cells (Fig. 2I,J) and other myeloid populations (Fig. 2K,L, gating strategy in Supplementary Fig. 1B). Together, this suggests that Jag1 expression in Tregs is dispensable for maintaining skin immune homeostasis.

### Jag1^pos^ Tregs are highly activated during early wound healing

One of the most common skin traumas is cutaneous injury. Treg depletion hinders both epithelial restoration^4^ and the full-thickness wound healing process^3^. One known function of Jag1^pos^Tregs is their role in facilitating hair follicle stem cell (HFSC) proliferation during hair growth phase transitions^1^. Therefore, we investigated whether Jag1^pos^ Tregs are involved in wound healing. Wound healing comprises four major overlapping stages: haemostasis, inflammation, proliferation, and remodelling^28^. Skin Treg abundance at full-thickness wound sites peaks at 7 days post wounding (dpw) and returns to homeostatic levels by 14dpw^3^. In Foxp3-DTR mice that permits systemic loss of all Tregs, delayed wound healing is observed only when Tregs are depleted during the inflammation phase, but not later. Jag1 expression in whole skin lysates follows a similar dynamic, being upregulated during the first 7dpw and downregulated from 14dpw^29,30^. Thus, we hypothesised that the first 7 days are likely to be a crucial period in which Jag1^Pos^ Tregs may have an impact.

**Fig. 3:**
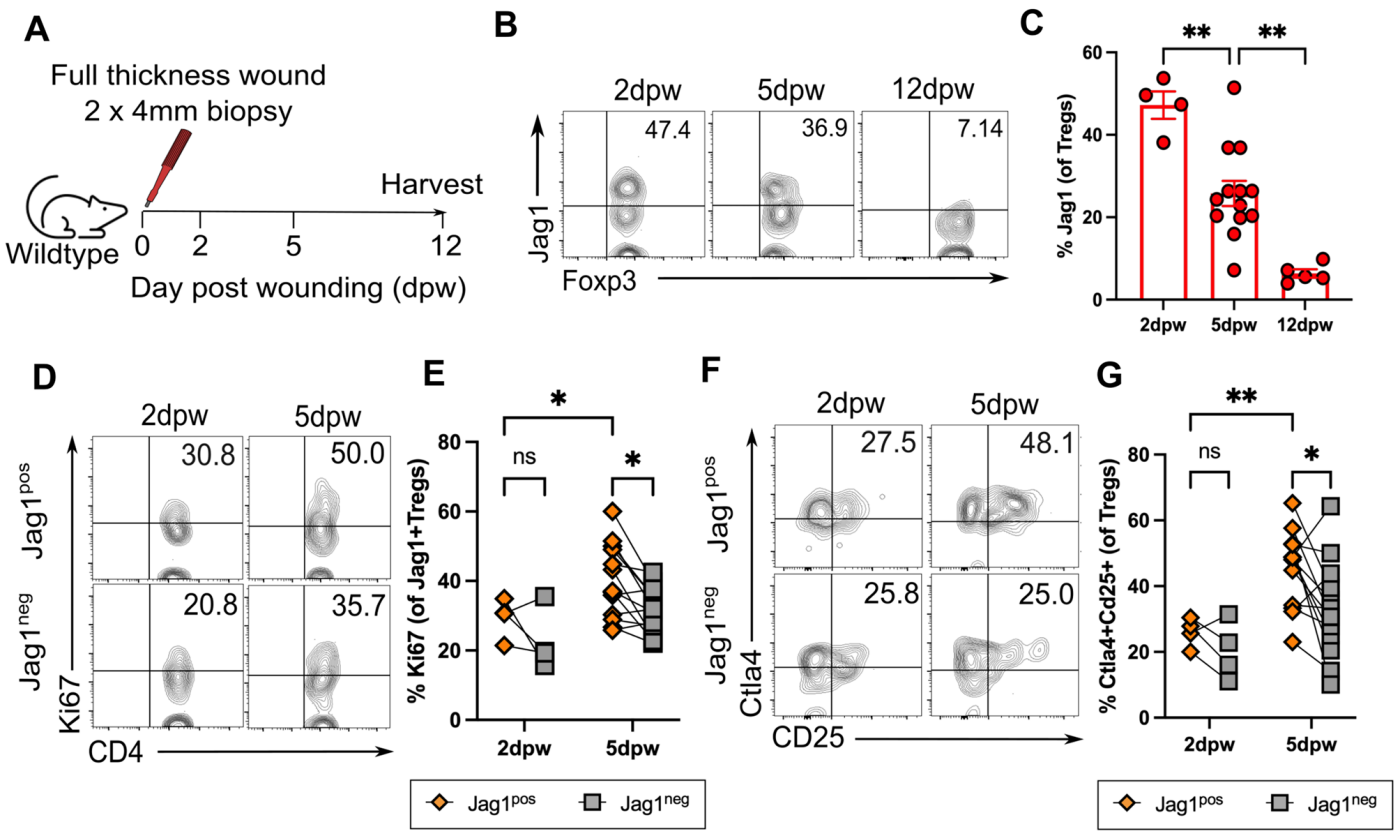
Jag1^Pos^ Tregs are most abundant and activated at 5 days post wounding (**A**) Experimental schematic in which two circular full thickness wounds were generated on the dorsum of wildtype mice using a 4 mm punch biopsy. Skin was harvested at 2 days post wound (dpw), 5dpw and 12 dpw. (**B**,**C**) Representative flow plots (**B**) and quantification (**C**) of % Tregs expressing Jag1 in wounded skin at 2dpw, 5dpw and 12dpw (n = 4–13 per group). Representative flow plots and quantification of (**D**,**E**) %Ki67 and (**F**,**G**) %CTLA4 + CD25 + in Jag1^Pos^ Tregs and Jag1^Neg^ Tregs from 2 and 5dpw wounded skin (n = 4–13 per group). Data in (**B**) to (**G**) were pooled from 2 independent experiments. Results were presented as individual data points with mean ± SEM in (**C**) and paired data-points in (**E**,**G**). Statistics were calculated by one-way ANOVA (**A**) and two-way ANOVA (**E**,**G**), *p < 0.05, **p < 0.01, ns = non-significant.

We first characterised the dynamic expression of Jag1^Pos^ Tregs by creating two 4 mm full-thickness wounds on the dorsum of wildtype adult mice at 0 days post wounding (dpw) and harvesting skin around the wound at inflammatory (2dpw), proliferative (5dpw) and remodelling (12dpw) phases (Fig. 3A). Notably, 25–48% of skin Tregs expressed Jag1 during the first 5 days post-wounding, in contrast to 6.35% (± 2.25%, *p* = 0.0014) at 12dpw (Fig. 3B,C). At 5dpw, Jag1^pos^Tregs were significantly more proliferative (Fig. 3D,E) and co-expressed higher levels of both CTLA4 and CD25 activation markers (Fig. 3F,G), relative to Jag1^neg ^Tregs. In contrast, no differences in Ki67 or CTLA4 ^+^ CD25 ^+^ proportions were observed between Jag1^pos^ and Jag1^neg^ skin Tregs at 2dpw, indicating that 5dpw is a critical period when Jag1^Pos^ Tregs’ may function.

### Jag1^Pos^ Tregs promote wound healing

**Fig. 4:**
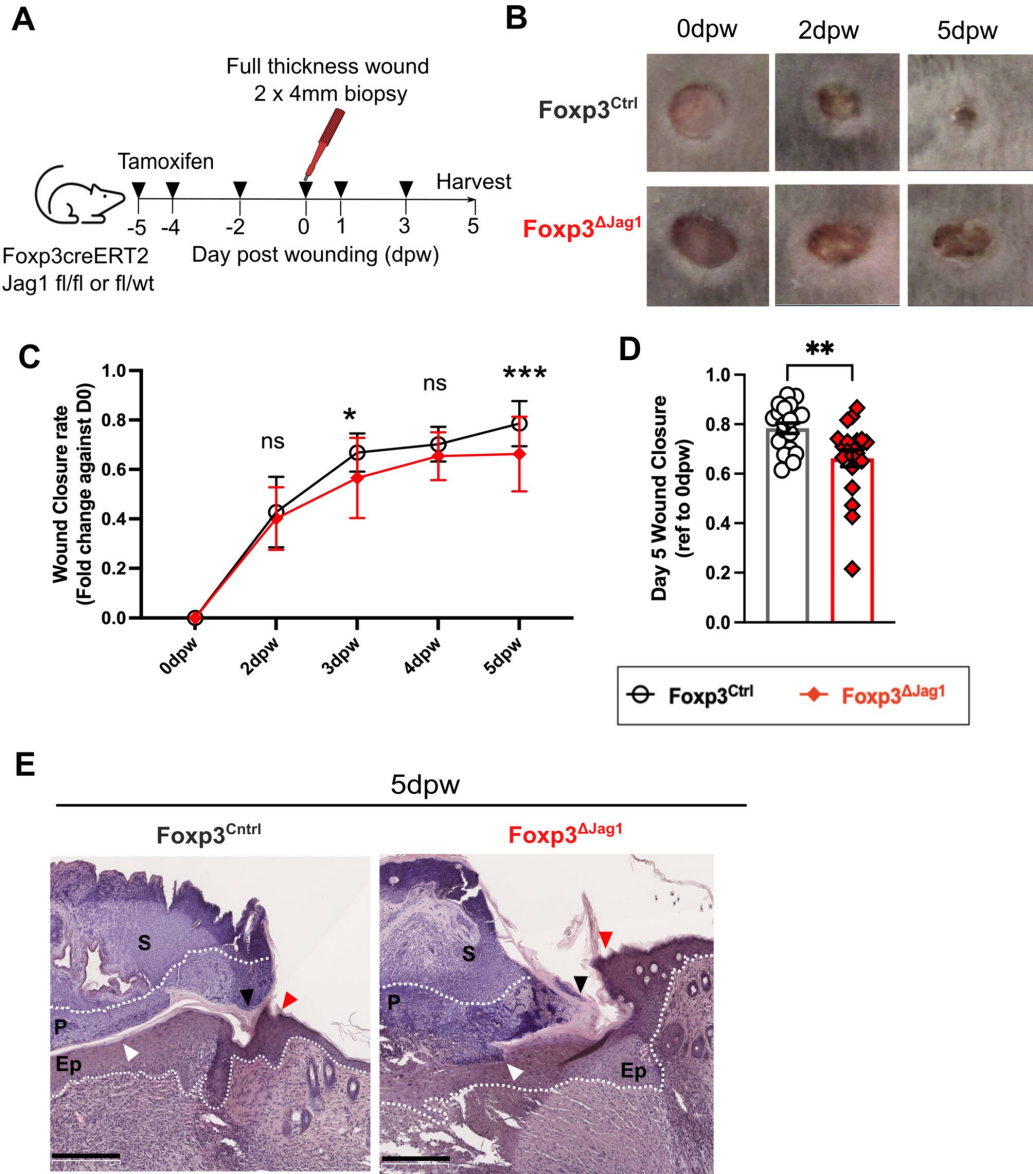
Jag1^Pos^ Tregs are required for wound healing. (**A**) Experimental schematic in which Foxp3^ΔJag1^ and Foxp3^Ctrl^ mice were injected with 4 doses of tamoxifen, before wounding with 4 mm biopsy punches. Two more doses of tamoxifen were given thereafter before wounded skin were harvested at 5dpw. (**B**) Representative clinical images of the healing progression at 0dpw (immediate after wounding), 2dpw, and 5dpw (**C**,**D**) Quantification of wound closure, calculated by fold change against wound area at 0dpw, with (**C**) showing the kinetics throughout the experiment, and (**D**) ratio at 5dpw (n = 20–22 per group). (**E**) Representative H&E image of Foxp3^Ctrl^ and Foxp3^ΔJag1^ wounded skin harvested at 5dpw, with labels of scab (S), pus cells (P) and epidermis (Ep). Arrows illustrated region of interest, including epidermal wound edge (red), keratinocyte precipitation (black) and border between pus cells and epidermis under scab (white). Scale bars represent 250 μm. (n = 4 per group). Data were pooled from 4 independent experiments. Results were presented as mean ± SEM in (C) and individual biological replicates as each data point with mean ± SEM in (D). Statistics were calculated by two-way ANOVA in (**C**), and unpaired t-test in (**D**).*p < 0.05, **p < 0.01, ***p < 0.001.

We then assessed the role of Jag1^pos^Tregs in wound healing by creating two full thickness excisional wounds of the dorsal skin of Foxp3^ΔJag1^ and Foxp3^Ctrl^ mice, and measured the wound healing rate over time (Fig. 4A). Mice lacking Jag1-expressing Tregs showed delayed wound closure compared to wildtype Foxp3^Ctrl^ mice treated with tamoxifen (Fig. 4B,C). The most pronounced difference was observed at 5dpw, with 78.4% (± 9.3%) wound closure in wildtype Foxp3^Ctrl^ mice versus 66.2% (± 15.9%) in Foxp3^ΔJag1^ animals (Fig. 4D, p = 0.0041). Histologically, Foxp3^ΔJag1^ skin showed a trend towards thicker epithelial wound edges (Fig. 4E, red arrow), more keratinocyte precipitation at wound edge (Fig. 4E, black arrow) and disorganisation between epithelial layer and pus cells (Fig. 4E, white arrow). These results illustrate skin Tregs require Jag1 for effective wound closure particularly during the early (5 dpw) proliferation phase.

### Jag1^Pos^ Tregs modulate neutrophil accumulation during early wound healing

We next explored the mechanisms by which skin Tregs use Jag1 to orchestrate wound closure. Upon tissue challenge, skin Tregs primarily perturb inflammation through two mechanisms: either via conventional immunosuppressive capacity^3,4^ or enhance tissue repair via cross-talk with epithelial cells and HFSCs^1,4^. Dysregulation of skin Tregs leads to unwanted inflammation, mainly contributed by excessive accumulation and proliferation of neutrophils, pro-inflammatory macrophages, CD4+ , and CD8+ T cells during homeostasis^20^, epidermal injury^4^, and full thickness injury^3^. Treg-specific loss of Rbpj, a downstream transcription factor of canonical Notch signalling, has been shown to mediate upregulation of Foxp3, CTLA4 and CD25 expression in splenic Tregs during homeostasis^19^. Given Jag1^pos^ Tregs co-express higher levels of CTLA4 and CD25 during both homeostasis and wound healing, relative to Jag1^neg^ Tregs, we questioned whether the absence of Jag1 in Tregs can affect the overall immunosuppressive ability of skin Tregs. We reasoned this may impact the accumulation of T and/or myeloid cells during wound healing and could influence wound closure through regulating local inflammation.

Similar to the steady state (Fig. 2), the absence of Jag1 in Tregs did not affect overall Treg abundance, as quantified by both flow cytometry and immunofluorescence staining (Supplementary Fig. 4A,B). Proliferation and Foxp3 expression in skin Tregs also remained unaffected (Supplementary Fig. 4C,D). No Treg phenotypic attributes were altered by loss of Jag1, including the proportion of skin Tregs co-expressing CTLA4 + CD25 + (Supplementary Fig. 4E), CD25 (Supplementary Fig. 4F) or ICOS (Supplementary Fig. 4G). IL33 is an alarmin highly induced in keratinocytes in response to cutaneous wounding^31^. IL33 receptor (ST2) is highly expressed in skin Tregs^10^, and is crucial for suppressing bleomycin-induced skin fibrosis^32^. However, we found that Tregs from Foxp3^ΔJag1^ skin did not have altered ST2 expression level (Supplementary Fig. 4H). Intriguingly, Jag1 ablation in Tregs resulted in a mild but significant reduction in CTLA4 levels (Supplementary Fig. 4I). Yet, both the accumulation and proliferation of Teffs and CD8+ T cells remained unchanged between Foxp3^ΔJag1^ and Foxp3^Ctrl^ skin (Supplementary Fig. 4J,K).

**Fig. 5:**
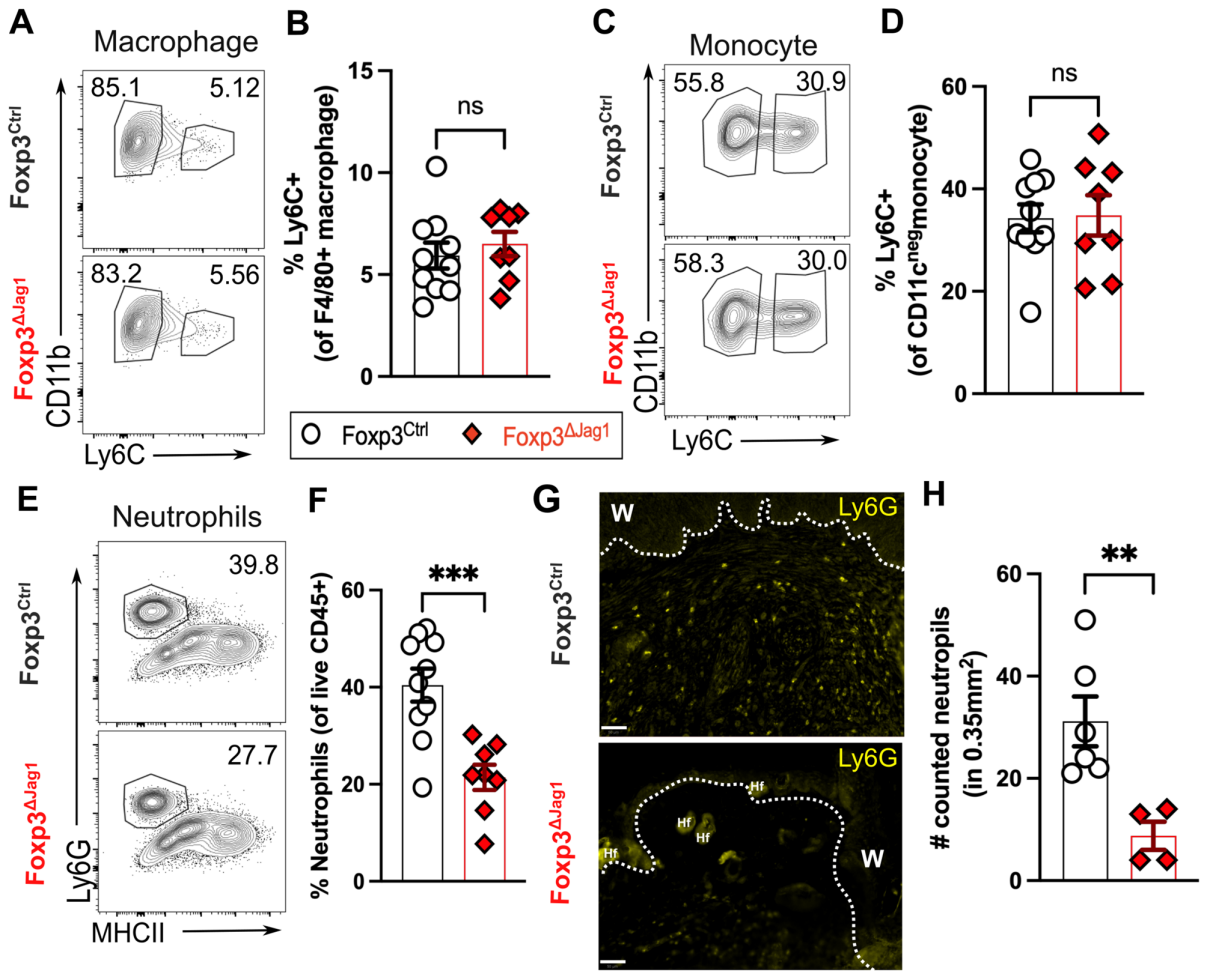
Jag1^Pos^ Tregs impact neutrophil accumulation in wounded skin. Representative flow plot and quantitation of (**A**,**B**) pro-inflammatory Ly6C + macrophages, (**C**,**D**) Ly6C + monocytes and (**E**,**F**) neutrophils in wounded skin at 5dpw (n = 8–10 per group). (**G**) Representative immunofluorescence staining of Ly6G of wounded skin, with labels of wound site (w) and hair follicle (Hf). Scale bars represent 50 μm. (**H**) Quantification of Ly6G + neutrophils from (**G**) within a fixed region-of-interest. (n = 4-6 per group). Data were pooled from 2 independent experiments. Each individual data point represented a biological replicate, and was collectively presented with mean ± SEM. Statistics were calculated by two-way ANOVA (**B**,**D** and **F**) and unpaired T-test (**H**). **p < 0.01, ***p < 0.001, ns = non-significant.

Innate cells are first responders to cutaneous wounding. Previously, it has been reported that pro-inflammatory Ly-6C^high^ macrophages accumulate at wound sites at 1 day post full-thickness wounding, contributing up to 60% of skin-resident macrophages, and gradually declining to around 10% at 7dpw^3^. Additionally, Treg depletion leads to a six-fold increase of Ly-6C^high^ macrophages at 7dpw, suggesting skin Tregs help transit the stages of wound healing from a pro-inflammatory to an anti-inflammatory environment. In contrast, Jag1 loss in Tregs did not lead to the same cellular skewing when compared to deletion of the entire Treg pool^3^. Instead, pro-inflammatory macrophage (defined as CD45 + CD11b^high^F4/80 + Ly-6C^high^Ly-6G^low^) accumulation in wounded skin remained indifferent between mice with Jag1-deficient and -sufficient Tregs, at around 6% of total macrophages at 5dpw (Fig. 5A,B). Similarly, the accumulation of inflammatory Ly6C + monocytes was unchanged (Fig. 5C,D), suggesting mice with Jag1-deficient Tregs remain capable of transitioning from a pro-inflammatory to an anti-inflammatory state during wound healing. Interestingly, the absence of Jag1 in Tregs led to less neutrophil influx into wounded skin, compared to wildtype controls at 5dpw, quantified by both flow cytometry and immunofluorescence staining (Fig. 5E–H). Collectively, Jag1 is unlikely to be a key factor driving the widely appreciated immunosuppressive function of skin Tregs. Rather, Jag1^pos^Tregs promote the retention of neutrophils during wound healing.

### Jag1^Pos^ Tregs do not influence re-epithelialization

Re-epithelialisation is a crucial step for successful wound healing following the inflammatory phase, by preventing excess water loss and further entry of microbial pathogens or debris. Trans-epidermal water loss measurement (TEWL) estimates moisture evaporation externally and is used quantitatively to assess epidermal integrity^33^. Besides suppressing inflammation, skin Tregs drive epidermal barrier repair by promoting bulge HFSC emigration to the epidermis, as well as their proliferation and differentiation^4^. Previous lineage tracing studies have shown that bulge cells repopulate the epidermis at 5 days post full thickness wounding^34^. These studies also indicate that bulge HFSCs are proliferative and can migrate to cutaneous wounds from 4dpw but not earlier^35^, and the derived cells can be detected in the epidermis for up to one year^36^. This indicates that while bulge HFSCs may not contribute to the immediate re-epithelisation of wounds, they play an important role in restoring the skin barrier from 4dpw onwards. Given that Jag1^pos^Tregs can drive bulge HFSC (identified as EpCam-Sca1-CD34 + CD49f+) proliferation during hair regeneration^1^, we hypothesised Jag1^pos^Tregs may also regulate wound closure by mediating bulge HFSC-driven re-epithelisation.

However, mice with Jag1 deficiency in Tregs showed a similar TEWL restoration rate to baseline as wildtype controls (Supplementary Fig. 5A), suggesting that Jag1^pos^Tregs do not influence barrier restoration post wounding. In line with this observation, the abundance and proliferation of bulge HFSCs also remained unaffected in Foxp3^ΔJag1^ mice during wound healing (Supplementary Fig. 5C,D, gating strategy in Supplementary Fig. 5B), indicating that Jag1^pos^Tregs are unlikely to share the same mechanistic interaction with bulge HFSCs as observed in hair regeneration.

## Discussion

Despite understanding the functional importance of skin Tregs, whether skin Tregs carry out their functions through the same molecular mechanisms as Tregs residing in other tissues is incompletely understood. One candidate is TGF-$\beta$ signalling, recently shown to drive both hair regeneration^2^ and epithelial barrier repair^5^. Yet, the functional importance of TGF-$\beta$ signalling extends globally to Treg generation and/or maintenance in both lymphoid and non-lymphoid tissues (Reviewed in^37^). Given that Jag1 is preferentially expressed in skin Tregs, we questioned whether Jag1-mediated signals are uniquely utilized by skin Tregs to drive their skin-related functions.

In the current study, we demonstrate that Jag1 is indeed preferentially expressed in skin Tregs. Jag1^pos^Tregs exhibit an activated profile, with upregulation of CD25 and CTLA4, but not ICOS, during both homeostasis and wound healing. Although it remains to be determined whether this upregulation reflects a higher immunosuppressive capacity of Jag1^pos^ Tregs, our survey of other non-Foxp3 expressing CD4+ , CD8+ T cells, pro-inflammatory macrophages, or monocytes, indicates skin Tregs are unlikely to suppress in vivo inflammation through Jag1.

Most notably, our study highlights the necessity of Jag1 expression in Tregs to facilitate adequate cutaneous wound repair. Consistent with previous findings on skin Tregs, the impact of Jag1^pos^ skin Tregs is limited to a critical time window (around 5 dpw) during which wound healing transitions from the inflammatory to the proliferation phase. However, rather than suppressing inflammation, Jag1 expression in Tregs appears to sustain the retention of the inflammation phase, as evidenced by neutrophil accumulation within the wounded environment.

The dual role of Jag1^Pos^ Tregs simultaneously promoting both wound healing and retention of neutrophils in the skin is not unprecedented, as neutrophils can exert both anti- and pro- inflammatory responses in response to acute tissue injury. Despite their well-established tissue-damaging properties, neutrophils are crucial facilitators in wound healing. Noted mechanisms include dissipation of infiltrating pathogens, damaged cells, and debris, and play an increasingly recognized role in resolving inflammation by polarizing anti-inflammatory macrophages, encouraging vascularization, and potentially promoting local cell proliferation to repair tissues (reviewed in^38,39^). We speculate that Jag1^Pos^ Tregs may produce neutrophil recruiting chemokines, such as CXCL5, CXCL8, or TGF1^40–42^, to promote either neutrophil accumulation or skew neutrophils towards adopting pro-reparative properties. For example, neutrophils utilize matrix metalloproteinase-9 signaling for collagen processing during lung repair^43^. In the skin, neutrophil-derived TNF-alpha can contribute to *Pseudomonas aeruginosa*-accelerated skin wound healing^44^.

To fully elucidate the mechanisms by which Jag1^Pos^ Tregs facilitate wound healing, future experiments will include comprehensive molecular profiling of wound-associated neutrophils in the presence and absence of Jag1^Pos^ Tregs, followed by neutrophil adoptive transfer studies to assess restoration of wound healing outcomes. These will include the same parameters we have tested here, namely clinical closure kinetics, immuno-profiling and analysis of epithelial stem and non-stem cell populations. However, there are many variables that we have not assayed in our study that can influence the rate of wound healing, such as age, gender, stress level, nutrition, wound hydration, and oxygenation rate (reviewed in^45,46^). With the increasing appreciation of how ageing could impact Treg prevalence and functional capacity, such as Tregs’ ability to drive neuron myelination^47^ or to direct muscle repair^48^, it is plausible that ageing could also affect skin Treg expression of Jag1 or their capacity to mediate wound healing.

Pertinently, mice lacking Notch activation in Lyz2 + myeloid cells (Lyz2^cre^RBPJ^fl/fl^) show a milder inflammatory response in the heart, lung and kidney upon lipopolysaccharide exposure^49^. This is also associated with a reduction in neutrophil accumulation, reduced inflammatory cytokine detection in the liver after injury^50^, and pro-inflammatory macrophage reduction in spinal cord lesion sites after compression injury^51^. This suggests that during injury, the activation of Notch signalling in myeloid cells can promote inflammation. Mechanistically, intrinsic Notch signaling plays an important regulatory role in dampening Tregs’ own immunosuppressive functions^19^. Our study also shows that despite high CTLA4 and CD25 expression both homeostatically and during wound healing, Jag1^pos^Tregs are unlikely to modulate intrinsic immunosuppressive capacity. While it remains to be elucidated whether Jag1^pos^Tregs directly influence Notch signalling in neutrophils, our study has uncovered an unexpected role for Jag1^pos^Tregs in modulating the inflammatory phase of cutaneous wound healing.

## Material and methods

### Animal study design

All mouse procedures were approved by local ethical approval at King’s College London (UK) (PP70/8474, establishment license X24D82DFF), and performed under a UK Government Home Office license (PP6051479). All methods were carried out in accordance with relevant guidelines and regulations under the UK animals (Scientific procedures) Act 1986, and were reported in accordance with ARRIVE guidelines. All possible efforts were made to minimize animal suffering. All experiments were performed on animals with no prior procedures. Animals were sacrificed using cervical dislocation, followed by permanent cessation of circulation as secondary.

Wildtype C57BL/6 and FoxP3^eGFP-CreERT2^ mice (JAX: 016,961**)** and Jag1^fl/fl^ (JAX: 010,618**)** from were purchased from the Jackson Laboratory J. FoxP3^eGFP-CreERT2^ were crossed to Jag1^fl/fl^ to generate FoxP3^eGFP-CreERT2^ Jag1^fl/fl^. Mice were maintained through routine breeding, were fed with a standard chow diet and housed in line with UK regulations. Littermates of the same sex were genotyped and assigned to experimental groups based on genotyping results.

Tamoxifen (Sigma, T5648-5G) were sonicated in 37 °C water bath and dissolved in corn oil, at 2.5 mg/ml concentration. For conditional Jag1 deletion, 7–10 week old Foxp3^eGFP-Cre-ERT2^Jag1fl/fl^fl/fl^ and Foxp3^eGFP-Cre-ERT2^Jag1^fl/wt^ or ^wt/wt^ were injected with tamoxifen intraperitonially at 75–100 mg/kg in the indicated interval before harvesting. All characterisation and steady state experiments were performed on mice of mixed gender, and only females were used for wound healing experiments.

### Skin wounding assays and analysis

Mice were anaesthetised by inhalation of vaporized 1.5% isoflurane, shaved and subcutaneously injected with Vetersgesics. Two full thickness excisional wounds were made on dorsal back of mice under anaesthesia, using a 4-mm biopsy punch (Stifel Laboratory Research). Wounds were photographed daily till harvest. The same ruler was placed next to wound area for measurement standardization. Wound area was measured using ImageJ^52^ (Fiji NIH), and closure ratio of each time point was calculated relative to wound area at 0dpw.

Transepidermal water loss (TEWL) of each wound was measured with Tewameter TM 300 probe (Courage + Khazaka electronic GmbH) according to the manufacturer’s protocols. Measurement were made on 0dpw (immediately after excision wound) and every 24 h thereafter. Each datapoint is an average of four TEWL measurements.

### Tissue processing

Whole murine dorsal skin was finely minced with scissors and digested in 500ul digestion medium per cm^2^ of skin. Digestion medium was prepared with 2 mg/ml collagenase (Sigma), 0.1 mg/ml DNase (Sigma) and 0.5 mg/ml hyaluronidase (Sigma), dissolved in C10 medium [10% FBS, 1% Pen/Strep, 1 mM Na-pyruvate, 1% HEPES, 1% non-essential amino acid, 0.5% 2-mercaptoethanol in RPMI-1640 with l-glutamine medium]. After 45 min incubation at 37 °C 255 rpm, single cell suspension was washed with 20 ml C10 medium, and filtered through 100um then 40um cell strainer. Lymph nodes were mechanically smashed, washed with FACS buffer [2% FBS, 1 mM EDTA in PBS], and filtered through 70um cell strainer. Epidermal cells were prepared by floating skin on 0.5%Trypsin–EDTA (Thermofisher) for 1 h at 37 °C, before gently removed from dermal part and washed with C10 medium. Lung was finely minced and digested with 1 mg/ml Collagenase A (Sigma) and 0.1 mg/ml DNase I (Sigma) in R10 [10% FBS and 1% Pen/Strep in RPMI-1640 medium]. After 1 h at 37 °C 180 rpm, single cell suspension is passed through 70um filter, spin for 5 min at 4 °C 1800 rpm, and treated with 500ul ACK Lysing Buffer for 30 s to 1 min before washed with 1xPBS. All single cell suspensions were then centrifuged at 1800 rpm at 4 °C for 4 min, and resuspended in 1 m FACS buffer. Total live cells were determined using NucleoCounter NC-200 (Chemometec) in 1:20 dilution, before downstream process.

For histology, skin tissue was fixed in 10% formalin overnight at 4 °C, followed by PBS washes, stored at 70% ethanol overnight at 4 °C, and embedded in paraffin using Epredia Excelsior tissue processor (Thermofisher).

### Tissue microarray

Each sample was cut and levelled to wound area, before manually assembled as tissue microarray blocks using a 4-mm biopsy punch. 5um sections were cut, mounted and sent to Tissueplexia (Scotland) for multiplex-staining using anti-FoxP3 (14-5773-82, Thermofisher) and anti-Ly6G (127,602, Biolegend) using previously published protocols^53,54^. H&E (Abcam) were performed according to manufacturer’s instruction and imaged using a Nanozoomer (Hamamatsu photonics) with a × 40 objective.

### Flow cytometry

For flow cytometry staining, 1.5–4 million cells per condition were plated in round bottom 96 well plate, and stained with 50ul of stated surface antibodies (see below) on ice for 20 min. After washed with FACS buffer, cells were fixed and permeabilised by FoxP3/Transcription Factor Staining buffer set (eBioscience) on ice for another 20 min, before washed with permeabilization buffer and lastly stained with intracellular antibodies (see below), again on ice for 20 min.

T cell panel was stained with anti-mouse, CD3 (Miltenyi, REAffinitiy and BioLegend, clone 17A2), TCRγ/δ (Miltenyi, REAffinitiy and BioLegend, clone GL3), CD4 (BioLegend, clone RM4-5), CD8a (Miltenyi, REAffinitiy, BioLegend clone 53–6.7), CD25 (Miltenyi, REAffinitiy, eBioscience, clone PC61.5), ICOS (BioLegend, clone C398.4A), Ki67(BD, clone B56), Foxp3 (Miltenyi, REAffinitiy and eBioscience FJK-16 s), CTLA4 (BD, clone UC10-4F10-11) and Jagged 1 (Santa Cruz, clone E-12 and eBioscience, clone HMJ1-29), Myeloid panel was stained with anti-Mouse Ly-6G (BD, clone 1A8), I-A/I-E (BioLegend, M5/114.15.2), CD45R/B220 (BioLegend, RA3-6B2), CD3 (BioLegend, clone 17A2), CD11c (BioLegend, clone N418), F4/80 (BioLegend, clone BM8), Ly6C (BioLegend, HK1.4), CD207 (eBioscience, eBioL31) and CD11b (BioLegend, M1/70). Epithelial panel was stained with CD326 (EpCam, Miltenyi REAfinity), Ki67 (BD, clone B56), CD49f (BD, clone GoH3), I-A/I-E (BioLegend, M5/114.15.2), CD34 (BD, RAM34) and Sca-1 (Miltenyi Reafinity).

All panels were stained with Zombie UV Fixable Viability kit (BioLegend) or GhostDye™ Live/Dead stain (Tonbo Biosciences) for live/dead distinction, followed by CD45 (eBioscience, clone 30-F11) for immune/non-immune cell separation. Samples were run on Fortessa LSRII (BD Bioscience) in KCL BRC Flow Cytometry Core. For compensation, UltraComp eBeads™ (Thermofisher) were stained with each surface and intracellular antibody following the same cell staining protocol. ArC™ Amine Reactive Compensation Bead Kit (Thermofisher, A10346) were used for GhostDye™ Live/Dead stain. All gating and data analysis were performed using FlowJo v10, while statistics were calculated using Graphpad Prism 10.

### RNA isolation and quantification PCR

Lymph node single cell suspension was resuspended in pre-sort medium [2% FBS, 1% Pen/Strep, 2 mM EDTA, 25 mM HEPES in RPMI-1640 without phenol red]. CD45 +CD3+CD4+CD25high (Tregs), CD45+CD3+CD4+CD25neg(Teffs) and CD45+CD3+CD8+ cells were sorted using FACSAria™ Fusion Flow Cytometer (BD) with 70/100um nozzle in KCL BRC Flow Cytometry Core. Cells were sorted into RPMI-1640 supplemented with 10% heat-inactivated FBS and 1% Pen/Strep, and spun at 300 g for 10 min at 4 °C. After removal of supernatant, cell pellet were snap-frozen in liquid nitrogen and stored at − 80 °C. RNA was extracted using NucleoSpin RNA XS, Micro kit for RNA purification (Macherey–Nagel) according manufacturer’s instruction. RNA integrity and concentration were then determined by RNA 6000 Pico Kit on Bioanalyzer (Agilent). RNA were then normalized and synthesied into cDNA using iScript cDNA synthesis kit (Bio-Rad). Quantitative PCR were performed using TaqMan™ PreAmp Master Mix Kit (ThermoFisher) on 384-well plate according to manufacturer’s instruction, run with 4 technical replica each condition. The following TaqMan probes were used: Gapdh (Mm99999915_g1, VIC), Foxp3 (Mm00475162_m1, FAM) and Jag1 (Mm00496904_m1, FAM), Plates were then run on CFX384 Touch Real-Time PCR system (BioRad).

### Quantification and statistical analysis

Parameters such as sample size, dispersion or precision are reported in Figure Legends. Statistical analyses were performed in Prism 10.1 (GraphPad). Details of the statistics and appropriate test used are also indicated in Figure Legends. *p < 0.05, **p < 0.01 ***p < 0.001, ****p < 0.001, *p*-values greater than 0.05 was identified as not statistically significant.

### Bulk RNAseq reanalysis

Read-count tables were obtained from NCBI database: GSE182322 (Ref^10^), and downstream processed according to presented method. Each data point in Fig. 1 represents the sum of both gene raw counts from ST2- and ST2 + Tregs per mouse.

## Supplementary Information

Supplementary Figures.

## Data Availability

The datasets used and/or analysed during the current study available from the corresponding author on reasonable request.
