# Supplementary material for: Jagged-1+ skin Tregs modulate cutaneous wound healing
Source: Sci Rep. 2024 Sep 9;14:20999. doi: 10.1038/s41598-024-71512-1 (PMC11385218; doi:10.1038/s41598-024-71512-1)
Supplement: Supplementary file 1 — Supplementary Figures. [file 41598_2024_71512_MOESM1_ESM.pdf]

Jagged-1+ Skin Tregs Modulate Cutaneous Wound Healing

Authors: Prudence PokWai Lui[1,2], Jessie Z. Xu[1,2], Hafsah Aziz[1,2], Monica Sen[1,2], Niwa Ali[1,2] *

Affiliations:
[1]Peter Gorer Department of Immunobiology, King's College London, London, UK
[2]Centre for Gene Therapy and Regenerative Medicine, King's College London, London, UK
*Corresponding author. Email: niwa.ali@kcl.ac.uk

SUPPLEMENTARY FIGURES

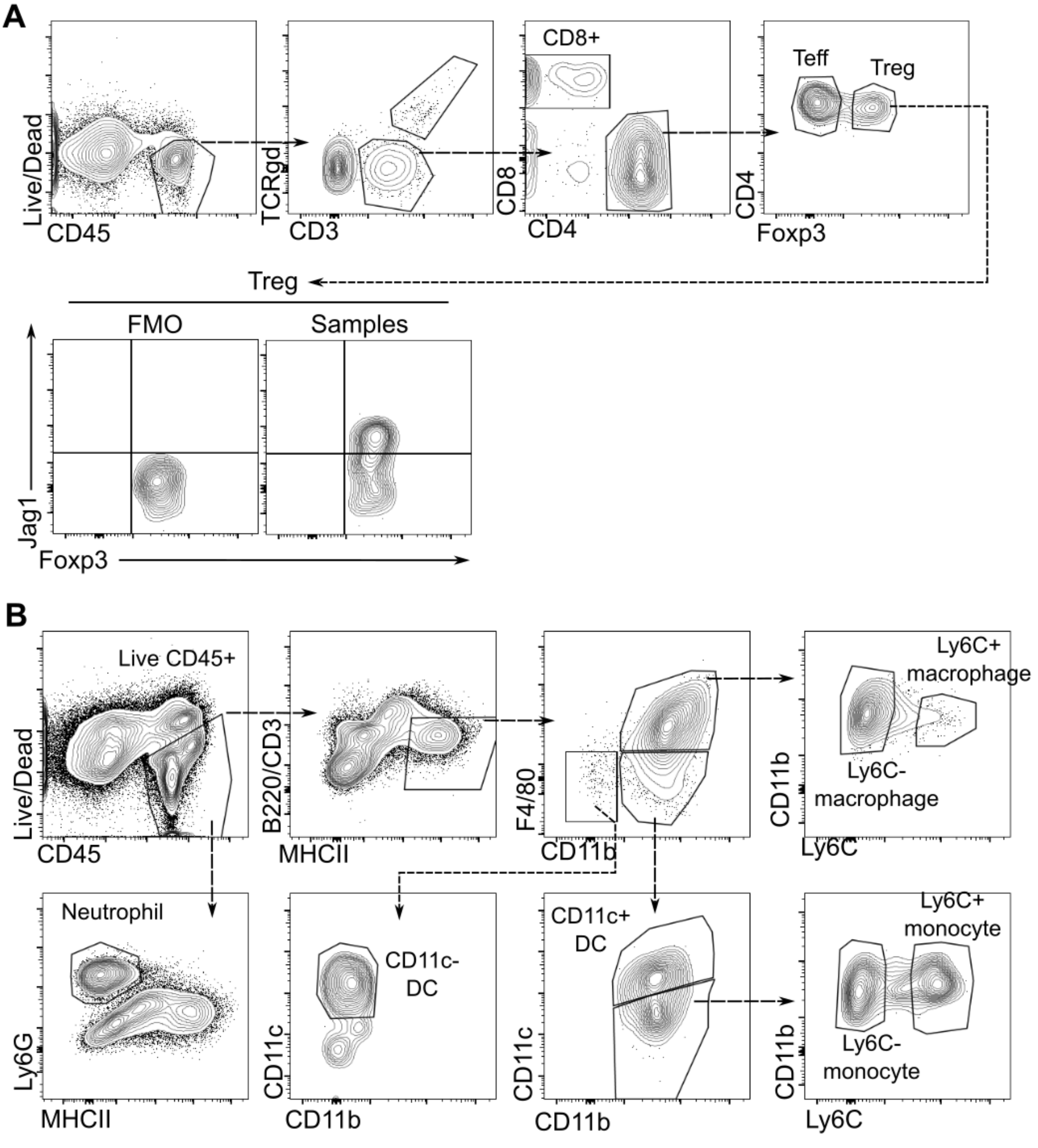

Supplementary Figure 1. Flow cytometric gating strategy to identify Jag1^{Pos} Tregs and skin T cell populations. Cells were first stained with Zombie UV live/dead stain and CD45 to identify live CD45. **(A)** T cell panel. T cells (TCRgd- CD3+) populations were further segregated based on CD8 and CD4 expression. Tregs were gated based on their Foxp3 expression. Jag1 gate was further determined based on FMO of all samples within each experiment. **(B)** Myeloid panel. Neutrophils (Ly6G+MHCII-) were gated directly from live CD45+ gate. B-cells and T-cells were excluded using B220 and CD3. Within the B220-CD3- MHCII+ gate, macrophages were identified as F4/80+ CD11b+ population, and were further separated based on their Ly6C expression. CD11b+CD11c- population was classified as monocytes, which was further subset into Ly6C+ and Ly6C- populations.

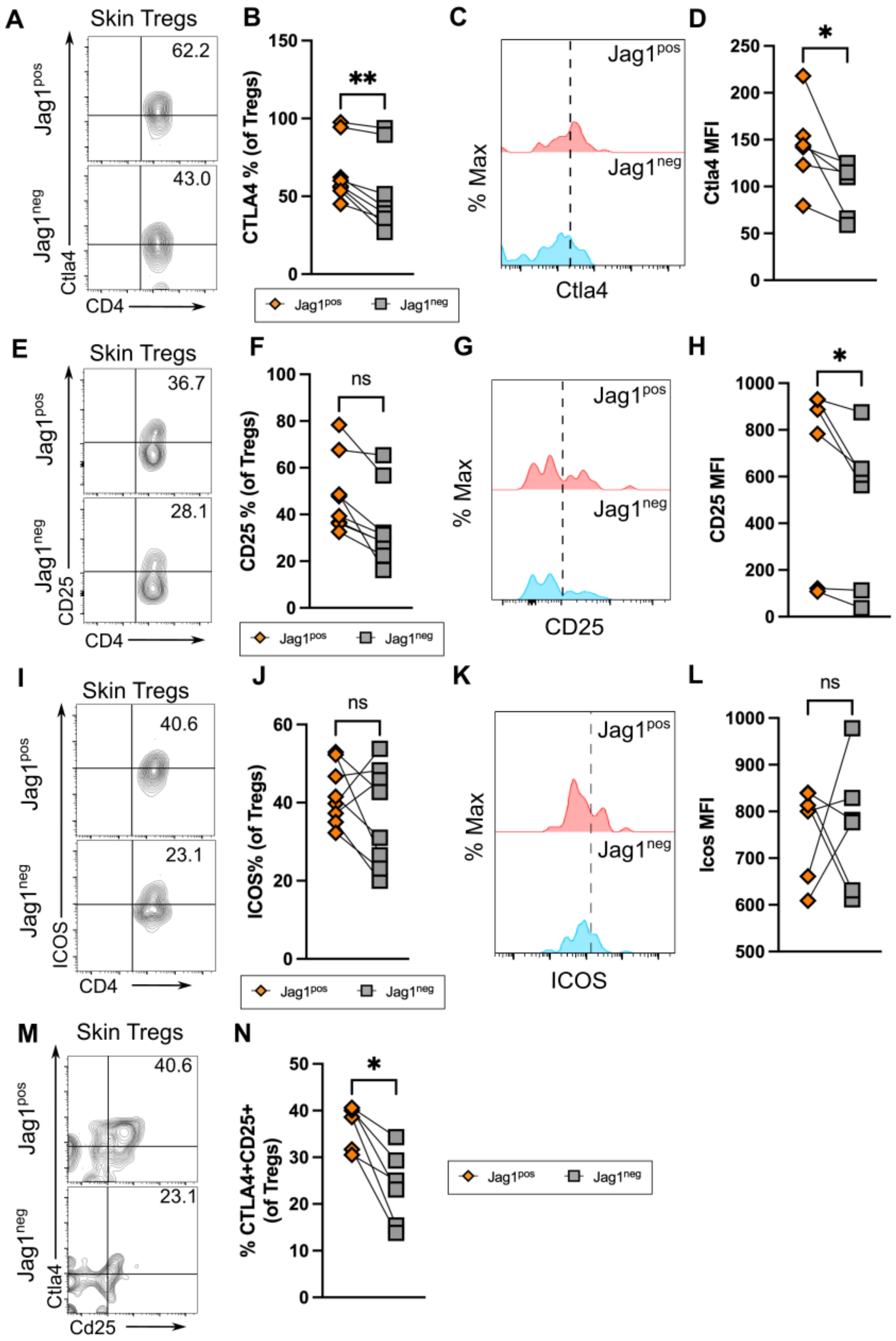

Supplementary Figure 2. Jag1^{Pos} Tregs are phenotypically more activated than Jag1^{neg} Tregs. Representative flow plot and quantitation of % Ctla4 (**A** & **B**), %CD25 (**E** & **F**), %Icos (**I** & **J**) and % Ctla4+Cd25+ (**M** and **N)** in Jag1^{pos} and Jag1^{neg} skin Tregs from wildtype

mice. Representative histogram and MFI quantification of Ctla4 (**C** & **D**), CD25 (**G** & **H**) and Icos (**K**& **L**) expressed in Jag1^{pos} and Jag1^{neg} skin Tregs. Dash line of histogram indicates gating from negative controls. Data were pooled from 2 independent experiments with n = 6. Each paired data point represented a biological replicate. Statistics were calculated by paired Wilcoxon t-test. * p < 0.05, ** p <0.01, ns = non-significant.

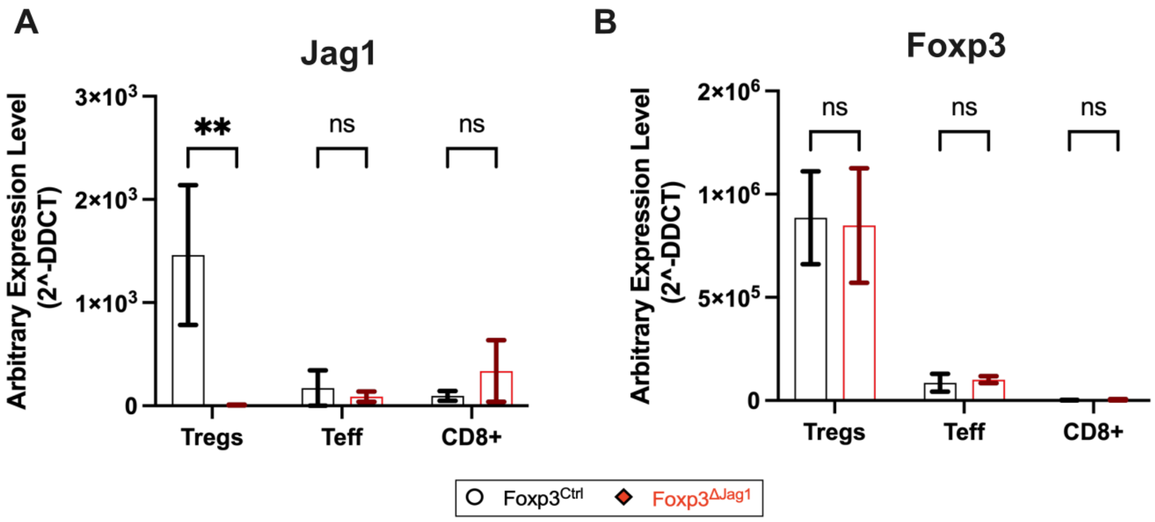

Supplementary Figure 3. qPCR of sorted Tregs to confirm Jag1 deletion in Tregs. Data were pooled from 2 independent experiments with n = 3-4. Results were presented as mean ± SEM. Statistics were calculated by two-way ANOVA, ** $p < 0.01$, ns = non-significant.

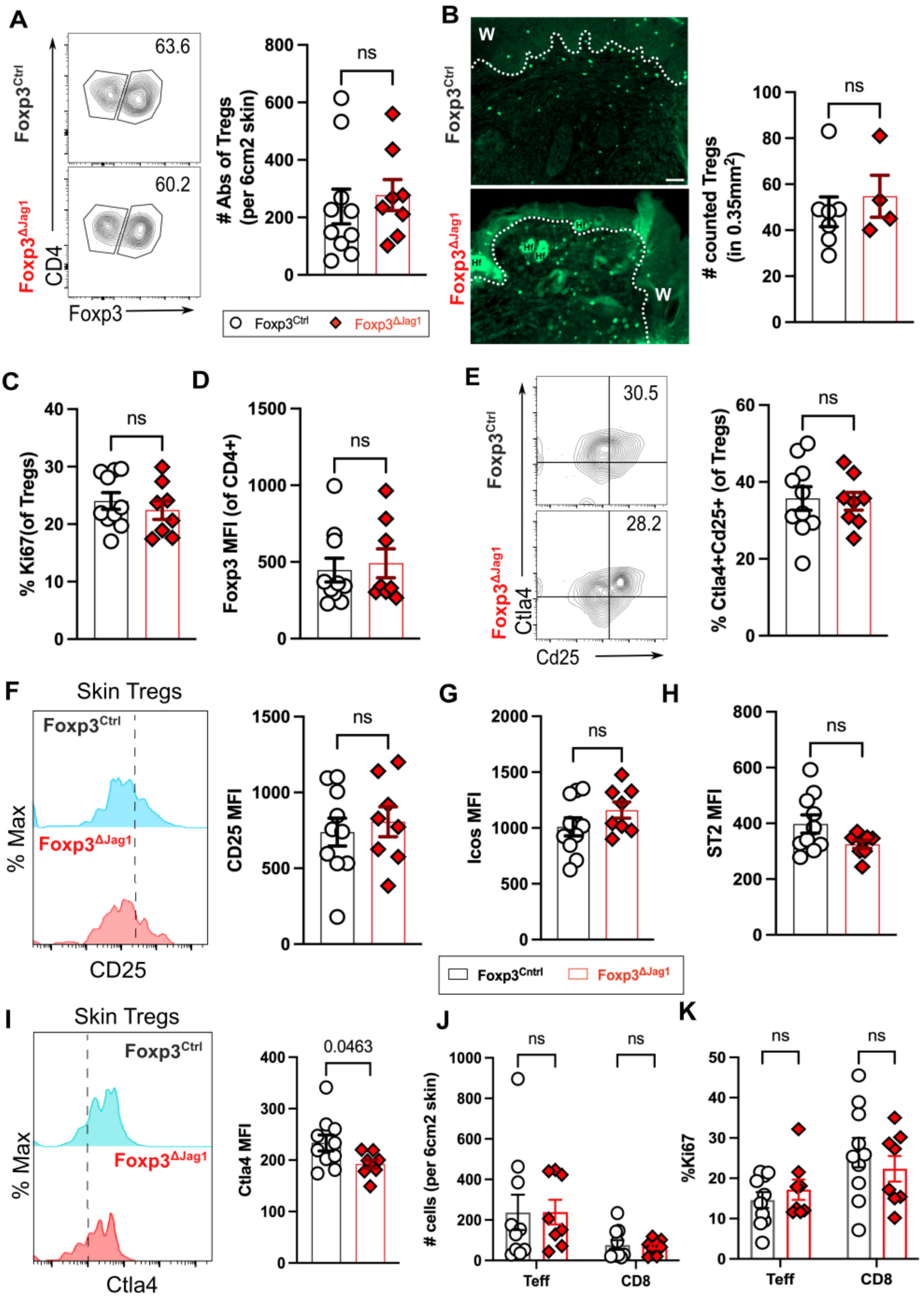

Supplementary Figure 4: Jag1^{Pos} Tregs do not influence T cell accumulation in wounded skin. (**A**)Representative flow plots and absolute abundance of Tregs from wounded skin at 5dpw (n = 8-10 per group). (**B**) Representative immunofluorescence staining and quantification of Foxp3 in wounded skin, with labels of wound site (w) and hair follicle (Hf). Scale bars

represent 50μm. (n = 4 per group). Flow cytometric quantification of (**C**) Treg %Ki67 and (**D**) mean fluorescence intensity (MFI) of Foxp3 in CD4+ T cells (n = 8-10 per group). Representative flow plot or histogram and quantification of (**E**) %Ctla4+CD25+, MFI of (**F**) CD25, (**G**) Icos, (**H**) ST2 and (**I**) Ctla4 in Tregs of wounded skin (n = 8-10 per group). Quantification of Teffs and CD8+ T cells **(J)** abundance, and **(K)** % Ki67 in wounded skin. Data were pooled from 2 independent experiments. Each individual data point represented a biological replicate, and was collectively presented with mean ± SEM. Statistics were calculated by unpaired T-test (A-I) and two-way ANOVA (J and K). ns = non-significant.

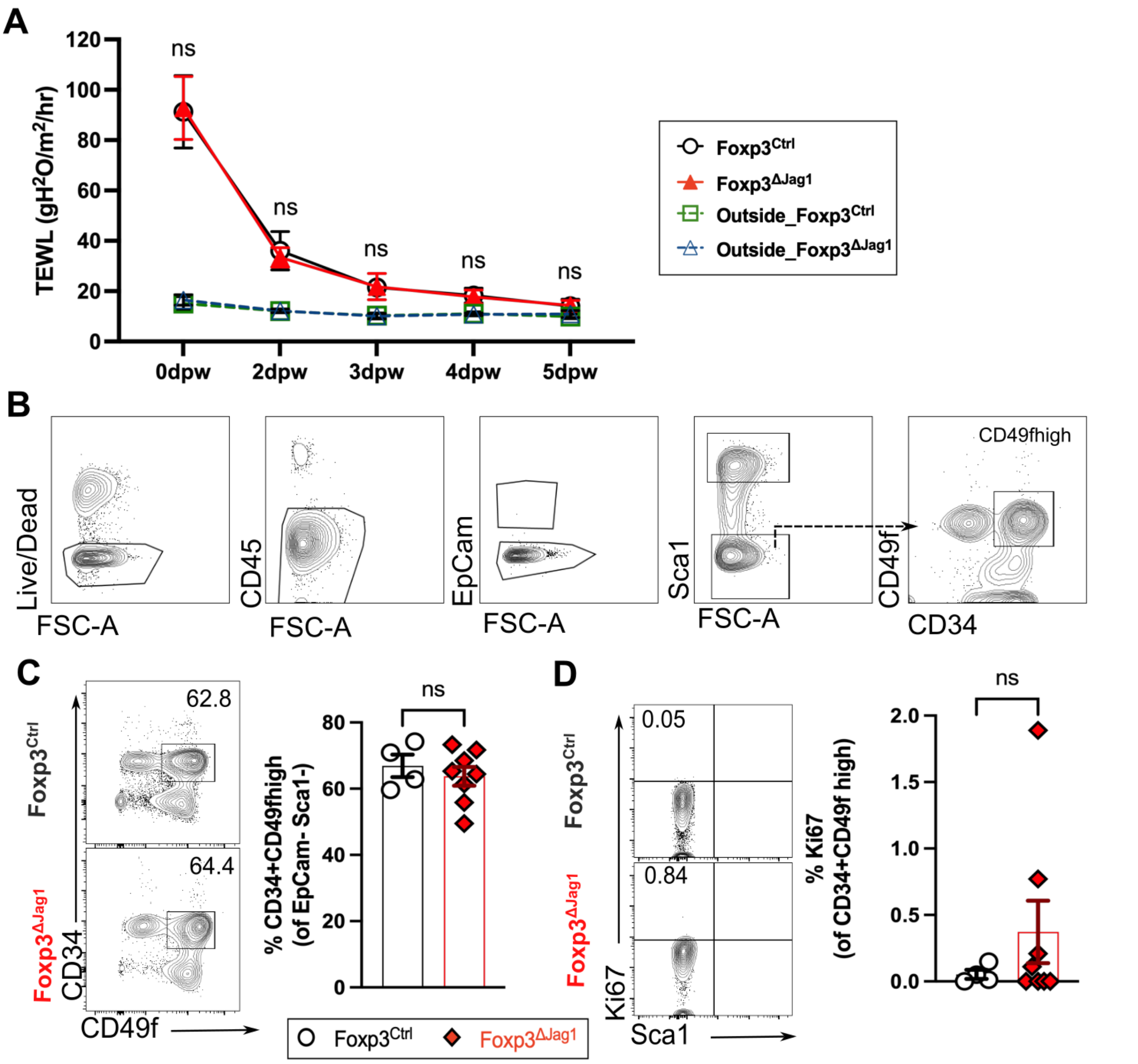

Supplementary Figure 5: Jag1^{Pos} Tregs do not influence HFSC homeostasis during wound healing. **(A)**Kinetics of trans epidermal water loss (TEWL) taken during wound healing of Foxp3^{Ctrl} and Foxp3$^{\Delta Jag1}$, with skin outside wound site setting the baseline of intact skin barrier (n = 8 per group).**(B)** Flow cytometric gating strategy to identify epithelial cell populations in skin. Cells were stained with Zombie UV live/dead stain and CD45 to identify live CD45neg populations. Epithelial cells were further separated into bulge HFSCs (Sca1+CD49f+CD34+). Representative flow plots and quantification of bulge HFSCs (**C**) abundance and (**D**) their proliferation from wounded skin at 5dpw (n = 4-8 per group). Data were pooled from 2 independent experiments. Each individual data point represented a biological replicate, and was collectively presented with mean ± SEM Statistics were calculated by two-way ANOVA (A) and unpaired T-test (C and D). ns = non-significant.
